# HIV-1 Tropism Determines Different Mutation Profiles in Proviral DNA

**DOI:** 10.1371/journal.pone.0139037

**Published:** 2015-09-28

**Authors:** Sieberth Nascimento-Brito, Jean Paulo Zukurov, Juliana T. Maricato, Angela C. Volpini, Anna Christina M. Salim, Flávio M. G. Araújo, Roney S. Coimbra, Guilherme C. Oliveira, Fernando Antoneli, Luiz Mário R. Janini

**Affiliations:** 1 Departamento de Microbiologia e Imunologia Veterinária, Universidade Federal Rural do Rio de Janeiro (UFRRJ), Rio de Janeiro, Brazil; 2 Departamento de Microbiologia, Imunologia e Parasitologia, Escola Paulista de Medicina (EPM), Universidade Federal de São Paulo (UNIFESP), São Paulo, Brazil; 3 Departamento de Medicina, EPM, UNIFESP, São Paulo, Brazil; 4 Genomics and Computational Biology Group, Research Center René Rachou (CPqRR), Fundação Oswaldo Cruz (FIOCRUZ), Belo Horizonte, Brazil; 5 Biosystems Informatics Group, CPqRR, FIOCRUZ, Belo Horizonte, Brazil; 6 Departamento de Informática em Saúde, EPM, UNIFESP, São Paulo, Brazil; 7 Laboratório de Biocomplexidade e Genômica Evolutiva, EPM, UNIFESP, São Paulo, Brazil; University Hospital Zurich, SWITZERLAND

## Abstract

In order to establish new infections HIV-1 particles need to attach to receptors expressed on the cellular surface. HIV-1 particles interact with a cell membrane receptor known as CD4 and subsequently with another cell membrane molecule known as a co-receptor. Two major different co-receptors have been identified: C-C chemokine Receptor type 5 (CCR5) and C-X-C chemokine Receptor type 4 (CXCR4) Previous reports have demonstrated cellular modifications upon HIV-1 binding to its co-receptors including gene expression modulations. Here we investigated the effect of viral binding to either CCR5 or CXCR4 co-receptors on viral diversity after a single round of reverse transcription. CCR5 and CXCR4 pseudotyped viruses were used to infect non-stimulated and stimulated PBMCs and purified CD4 positive cells. We adopted the SOLiD methodology to sequence virtually the entire proviral DNA from all experimental infections. Infections with CCR5 and CXCR4 pseudotyped virus resulted in different patterns of genetic diversification. CCR5 virus infections produced extensive proviral diversity while in CXCR4 infections a more localized substitution process was observed. In addition, we present pioneering results of a recently developed method for the analysis of SOLiD generated sequencing data applicable to the study of viral quasi-species. Our findings demonstrate the feasibility of viral quasi-species evaluation by NGS methodologies. We presented for the first time strong evidence for a host cell driving mechanism acting on the HIV-1 genetic variability under the control of co-receptor stimulation. Additional investigations are needed to further clarify this question, which is relevant to viral diversification process and consequent disease progression.

## Introduction

HIV-1 adsorption to host cell depends on the ligation to cell membrane receptor, CD4, and the subsequent penetration requires interaction with another host cell membrane molecule known as a co-receptor. Two major different co-receptors have been identified: C-C chemokine Receptor type 5 (CCR5) and C-X-C chemokine Receptor type 4 (CXCR4) [1, 2]. These cellular molecules belong to the G-protein coupled receptors superfamily, and are primarily involved in the activation of neutrophils (CXCR4), monocytes, lymphocytes or basophils (CCR5) [3]. Both co-receptors are expressed in T CD4 positive lymphocytes, the main target cell for HIV-1, being CCR5 preferentially present in the effector memory T cells subset, whereas CXCR4 is abundant at the surface of naive T cells [4]. Macrophages, another important HIV-1 target cell, also express both co-receptors being CXCR4 less abundant [5]. The glycoprotein 120 (gp 120), a virus surface protein encoded by the *env* gene, is the viral binding component to the cellular receptor and co-receptor. It contains conserved (C1-5) and variable (V1-5) regions. Scattered amino acid positions in the primary sequence of conserved regions are directly implicated in the contact to CD4 receptor [6]. The variable region 3 (V3 loop) of gp 120 is involved in co-receptor binding, and changes in its amino acid composition are responsible for phenotypic changes in the co-receptor usage [7].

Regarding to the use of co-receptors, three different phenotypes are recognized in HIV-1: (i) R5, for CCR5 employing viruses; (ii) X4, for CXCR4 employing viruses; and (iii) R5X4 dual-tropic, for both co-receptors employing viruses. Usually, R5 viruses are found at early stages of infection and X4 viruses are more often related to T CD4 positive cells decay and AIDS development [1–3]. According to literature different cellular outcomes after CCR5 or CXCR4 stimulation by the attachment of R5 or X4 viruses have been described [8–10]. R5 viruses have been related to the stimulation of genes related to the cell cycle regulation. Some authors also reported that cellular transcriptional modulations are mostly a consequence of the interaction between the virus and its co-receptor rather than to the binding to CD4 receptor itself [9]. Another recent report showed a higher capability of R5 viruses to induce a specific host DNA repair mechanism involving a dUTP removing enzyme, when compared to X4 viruses [11]. This activity could counteract the effect of APOBEC, thus reducing the impact of hypermutation on HIV genome [12, 13].

Mutation is an important driving force during the evolution of biological entities, particularly for RNA viruses to which the quasi-species concept has been applied. HIV-1 is such an example [14]. Since a *quasi*-species is defined as a spectrum of mutant virus acting as a whole, a populational approach is suitable in order to evaluate its biological properties. Next generation sequencing (NGS) methods had been proposed as an analytical tool in virology [15, 16], and are employed in HIV-1 studies as exemplified in some recent publications [17–19]. However, technical peculiarities have made some methods more commonly used than others [20]. As an example, SOLiD sequencing methodology (Life Technologies) has been used only in specific cases when haplotypes reconstruction is not believed to be mandatory [21, 22]. The short length reads generated by SOLiD are not adequate for individual genome reconstruction. On the other hand according to the quasi-species theory the ensemble of variants is much more relevant than a specific viral genome [14]. Consequently SOLiD is suitable for the study of viral quasi-species. To our knowledge, that methodology has never been proposed to evaluate viral populations without the need to track individual genomes.

Here we present the results of nucleotide substitutions occurring in proviral DNA populations after a single round of reverse transcription in cultures of primary infected cells with either R5 or X4 pseudotyped viruses. Pseudotyped viruses were used to infect resting and stimulated PBMCs and purified CD4 positive cells. We also compared the influence of target cell homogeneity and its physiological status, i.e., PBMCs versus purified CD4 positive cells, and resting versus stimulated cells on the observed viral diversity. We used the SOLiD methodology to sequence virtually the entire proviral DNA from each experiment.

We considered the aligned sequence reads, obtained from each infection condition, as representative of the HIV-1 provirus population after a single replicative event. Also, we present in this paper the first results of a recently developed method for the analysis of SOLiD generated sequencing data devoted to the study of viral quasi-species [23].

## Results

### Host cells preparation and infection assay

**Fig 1:**
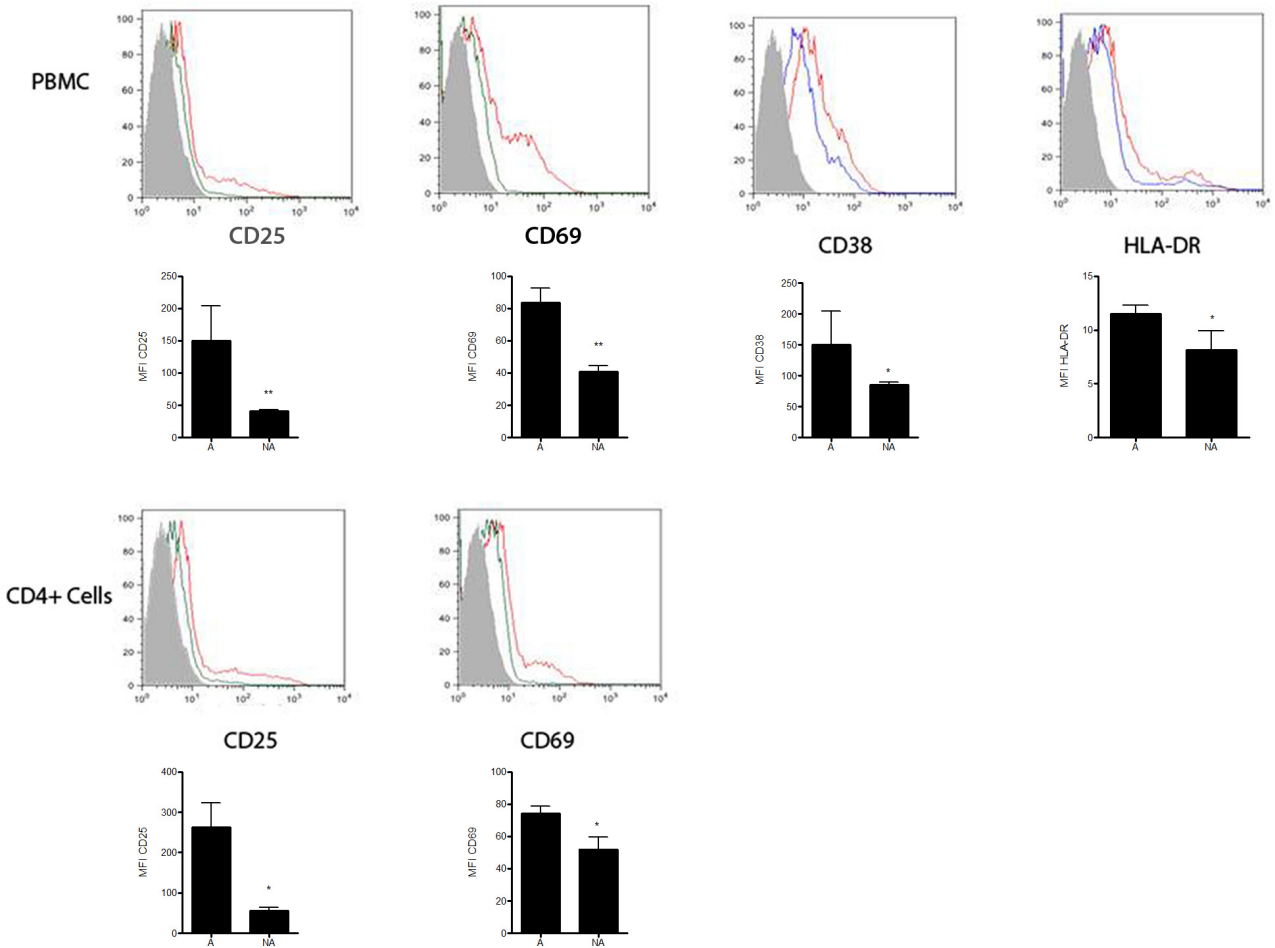
Agarose gel with amplified products from the CCR5 gene from donor’s infected cells showing the absence of Δ 32 deletion. Lane **A:** molecular size marker, lane **B:** amplified region of the CCR5 gene from donor’s cells, used in the study lane **C:** homozygous wild type control showing the expected wild type band control containing 241 bp, lane **D:** heterozygous wild type/Δ32 control.

**Fig 2:**
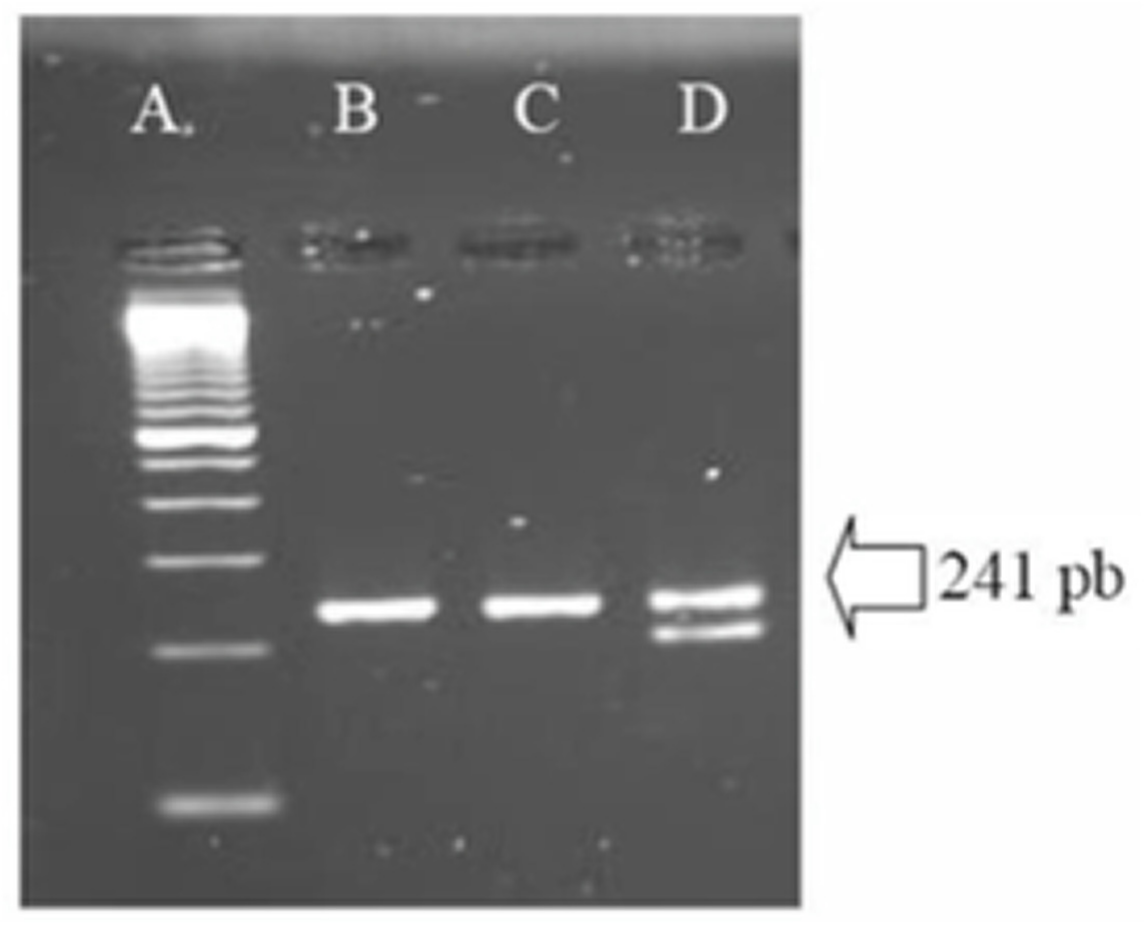
Expression of Activation Markers using flow cytometry assays. Histograms showing the expression of CD25, CD69, CD38 and HLA-DR from gated CD3+CD4+ cell populations from PBMCs are shown in the upper row. Histograms indicating the expression of CD25, CD69 from gated CD3+CD4+ cell populations from purified T CD4+ cells appear in the lower row. X axis indicates the fluoresce intensity of a specific marker and Y axis indicates the percentage of cells in the population expressing this specific marker. Graphics under each histogram represent the MFI (Median of Fluorescence Intensity) of each activation marker in the gated CD3+CD4+ cell populations. Results are shown as mean values ± SD and are representative from three independent experiments. Statistical significance was assessed by the Two-tailed Student’s t-test yielding * when p < 0.05; and **, when p < 0.005. Gray area: unstained control cells, green/blue line: non-stimulated cells, red line: stimulated cells.

A total of 1.2 x 10^8^ viable PBMCs/mL were obtained from a single seronegative donor. Part of those cells was further used for selection of CD4 positive cells. In order to access the Δ32CCR5 deletion in those cells, an aliquot of PBMCs was used for PCR amplification of the CCR5 coding gene. Fig 1 shows the wild type homozygous state assuring those cells could be infected by R5 HIV-1 variants. Part of each group of cells was stimulated as described in the Methods section, and the stimulation of cells was indirectly assessed by flow cytometry assay (Figs 1 and 2). Resting state was suggested by the low density or absence of phenotypic lymphocyte activation markers at the surface of non-stimulated cells, allowing us to conclude that these cells were at the G0 phase (quiescent state) of the cell cycle [24]. On the other hand, stimulated cells showed an increase in lymphocyte activation markers. Differences in the expression of activation markers before and after stimulation were assessed by MFI (Median of Fluoresce Intensity). Values of MFI for all tested markers were significantly different (p values bellow 0.05) between unstimulated and stimulated cells. Pseudotyped HIV-1 viruses were harvested from the supernatant of transfected HeLa cells. A mean value of 1.3 x 10^7^ viral particles/mL for pseudotyped viruses (X4 and R5) were obtained and infections of blood derived cells performed. After 72 hours of infection no evidence of cytopathic effects was observed for neither pseudotyped viruses. Also no virus was detected in the supernatant of all primary cultures after pelleting down infected cells (see Methods section).

### Proviral DNA harvesting, sequencing and data analysis

The successfully amplification of the almost complete proviral sequence from infected cells were achieved for every virus-cell combination, except for stimulated purified CD4 positive cells infected by X4 pseudotyped viruses. In this experimental condition we could obtain two out of four expected proviral regions, according to the amplification strategy presented in Methods section. In order to maintain homogeneity of sample results we decided to exclude that experimental non-amplified condition from the present study. No amplicons were obtained from uninfected PBMCs or T CD4 positive purified cells. Both pseudotyped viruses (R5 and X4) had the same genome which was derived from the pNL4-3kfs plasmid. This plasmid was the sole source of genomic template DNA during virus production (see Methods section).

**Fig 3:**
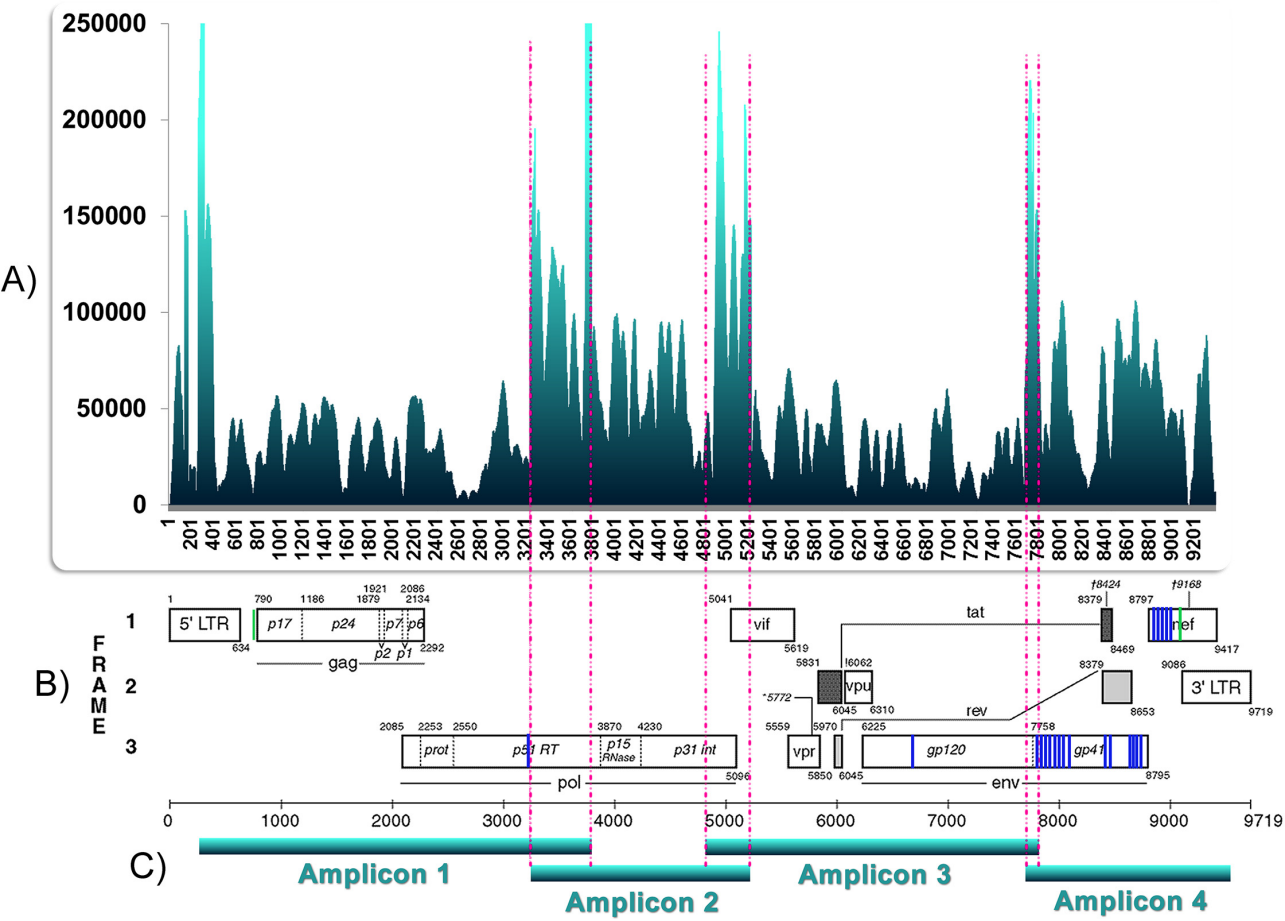
Depth coverage of SOLiD sequencing per nucleotide position, observed nucleotide changes per position and amplification strategy. **A)** X axis indicates positions along the HIV-1 genome and Y axis the number of times each position was sequenced. Shown here the sequence depth coverage obtained from stimulated purified CD4 positive cells infected by R5 pseudotyped virus. **B)** Nucleotide changes affecting the majority of sequences from the proviral population. Data obtained from non-stimulated purified CD4 positive cells infected with X4 pseudotyped HIV-1 after a single round of reverse transcription. Diagrammatical location of changes: blue lines = mutated positions; green lines = boundaries of the investigated region. **C)** Amplification strategy was performed with 4 overlapping sub-genome fragments (A,B,C,D).

In order to apply our analysis methodology was mandatory to include viral sequences obtained from the pNL4-3kfs plasmid generated by the SOLiD methodology. This data was used as the initial viral genome condition prior to infections. Amplified proviral DNA from all infections was also sequenced in the SOLiD v.3 platform (Life Technologies) and the raw data was analyzed as previously described [23]. Table 1 presents the number of validated reads and sequenced nucleotides for each experimental condition and control. Reads mapping to the 5’ or 3’ LTR regions were concentrated at either one of them during data analysis because of sequence homology, resulting in exceptional depth coverage. For this reason, we retained for posterior analysis only the region comprised between nucleotide positions 791 to 9,085, taking the HIV-1 NL4-3 genome as reference [GenBank accession number AF324493]. Validated reads were used to create a populational picture of the sequenced DNA molecules instead of reconstructing haplotypes present in each experimental condition [23]. Table 2 shows the base composition of the total proviral DNA from each infection and from the initial genome (pNL4-3kfs). Table 3 indicates the number of times each proviral genomic position was sequenced (depth of coverage). All positions were covered to a great depth (min. 584, max 328,621 times). Fig 3A presents an example of the depth sequencing coverage distribution per nucleotide position. The observed peaks are related to artifactual or expected accumulation of reads in selected proviral regions. 5’ and 3’ LTR peaks are assumed to be artifactual, whereas those present at other regions are assumed to be due to the overlapping of amplicons during the sequencing reactions (Fig 3C). Similarly, pronounced decreases in depth coverage at LTR regions, with nearly no mapped reads, are due to the concentration of all LTR reads at one LTR region.

**Table 1:** Number of sequenced reads (50 nucleotide long in average) and of nucleotides after SOLiD sequencing per experimental condition.

Experimental condition	n° reads	n° sequenced nucleotides
**Stimulated CD4 + cells/ R5 HIV-1**	1.04 x 10^7^	5.19 x 10^8^
**Non-stimulated CD4 + cells/ X4 HIV-1**	1.10 x 10^7^	5.50 x 10^8^
**Non-stimulated CD4 + cells/ R5 HIV-1**	1.11 x 10^7^	5.53 x 10^8^
**Stimulated PBMC/ X4 HIV-1**	9.41 x 10^6^	4.71 x 10^8^
**Stimulated PBMC/ R5 HIV-1**	1.14 x 10^7^	5.73 x 10^8^
**Non-stimulated PBMC/ X4 HIV-1**	1.12 x 10^7^	5.62 x 10^8^
**Non-stimulated PBMC/ R5 HIV-1**	1.19 x 10^7^	5.94 x 10^8^
**pNL4-3kfs control**	1.01 x 10^7^	5.05 x 10^8^
**TOTAL**	8.65 x 10^7^	4.33 x 10^9^

**Table 2:** DNA base content (A, T, C, G) per experimental condition taken into account all sequence reads *

	Stimulated CD4 + cells/ R5 HIV-1	Non-stimulated CD4 + cells/ X4 HIV-1	Non-stimulated CD4 + cells/ R5 HIV-1	Stimulated PBMC/ X4 HIV-1	Stimulated PBMC/ R5 HIV-1	Non-stimulated PBMC/ X4 HIV-1	Non-stimulated PBMC/ R5 HIV-1	pNL4-3kfs control
**A**	36.3	35.6	35.6	35.7	35.9	36.4	36.1	37.2
**T**	21.5	20.7	20.8	21.1	21.0	21.2	21.1	21.6
**C**	17.3	18.0	18.0	17.8	17.9	17.4	17.7	17.1
**G**	25.0	25.7	25.7	25.4	25.2	25.0	25.1	24.1

*% Percent numbers

**Table 3:** Depth coverage of SOLiD sequencing per experimental condition covering positions 790 to 9085 of the HIV-1 pNL4-3 reference genome*.

Experimental condition	Mean depth	Maximal depth	Minimal depth
**Stimulated CD4 + cells/ R5 HIV-1**	53,686.49	264,854	808
**Non-stimulated CD4 + cells/ X4 HIV-1**	48,013.81	307,51	1,145
**Non-stimulated CD4 + cells/ R5 HIV-1**	51,874.62	328,621	584
**Stimulated PBMC/ X4 HIV-1**	45,543.92	251,851	921
**Stimulated PBMC/ R5 HIV-1**	54,555.82	296,26	1,183
**Non-stimulated PBMC/ X4 HIV-1**	54,194.89	309,272	630
**Non-stimulated PBMC/ R5 HIV-1**	58,211.36	308,238	1,174

*HIV-1 pNL4-3 reference genome [GenBank accession number AF324493]. Presented data correspond to number of reads obtained from each condition.

The statistical inference implemented in our analysis method as described previously [23], provided a probability of occurrence of each nucleotide per genomic position, as exemplified in Table 4 (for the probabilities at all sites on the seven experimental conditions and its control see S1 Data). The probability of occurrence of each of the 4 possible nucleotides at a position was calculated based on the total generated and validated reads covering this same position. This probability was calculated for each infection and for the initial viral genome (pNL4-3kfs).

**Table 4:** Example of six nucleotide probabilities per position obtained from non-stimulated purified T CD4 positive cells infected by X4 pseudotyped HIV-1*.

Nucleotide position	Nucleotides probabilities	Nucleotide position	Nucleotides probabilities
**7764**	C (98.187)	**7875**	A (83.166)
	A (01.714)		C (16.685)
	T (00.087)		G (00.129)
	G (00.012)		T (00.020)
**7765**	T (98.915)	**7876**	T (54.227)
	G (00.661)		C (45.667)
	C (00.410)		G (00.096)
	A (0.014)		A (00.010)
**7766**	T (80.621)	**7877**	T (52.707)
	A (18.371)		C (45.421)
	C (00.639)		A (01.611)
	G (00.369)		G (00.261)

*Numbers in parenthesis indicate the nucleotide probabilities as percentages at the specified positions.

Comparing the above mentioned probabilities at every proviral position with the corresponding probability at each nucleotide position in the initial genome (pNL4-3kfs) enabled us to identify a pattern of nucleotide probability differences after one round of reverse transcription. Nucleotides were ranked from the most probable to the least probable for each sequence position. By doing this, we were able to establish two categories of changes for each position: (i) changes in which nucleotides appeared at the same ranking order from most probable to least probable, but with differences in each nucleotide probability and (ii) changes in which the nucleotide order changed, mainly involving the most probable nucleotide among the sequenced population from each experiment. We classified those categories of changes as quantitative and qualitative, respectively.

**Fig 4:**
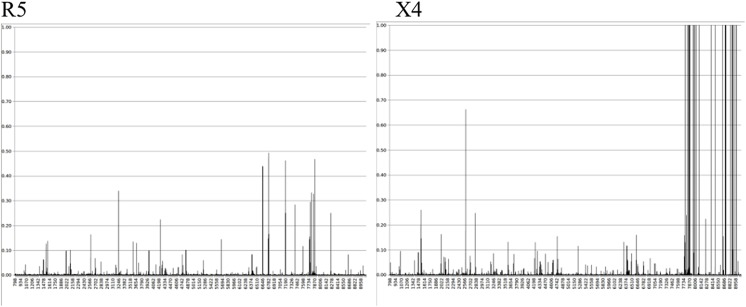
Representative range of variational distances between nucleotide probabilities observed in the control and in each experimental condition. X axis represents nucleotide positions in the sequence and Y axis indicates variational distances. Left, infection of purified non-stimulated T CD4 positive cells by R5 pseudotyped HIV-1; right, infection of non-stimulated PBMCs by X4 pseudotyped HIV-1.

The observed changes in the afored mentioned probabilities were strongly influenced by the pseudotyped virus tropism, as exemplified in Fig 4. Probability differences also referred as variational distance was pronounced at the *env* region during X4 tropic pseudotype viruses infections. However, differences in the same range of magnitude were not observed when R5 tropic pseudotyped viruses infected either PBMCs or purified non stimulated CD4 positive cells. Changes at the *env* region observed for X4 pseudotyped HIV-1 were reproduced at every X4 infection condition (including infections of unstimulated and stimulated PBMCs and T CD4+ cells. Similarly every experiment with R5 pseudotyped HIV-1 produced R5 equivalent results.

### Host cell type and activation status did not significantly affect the nucleotide probabilities in proviral sequenced populations

**Fig 5:**
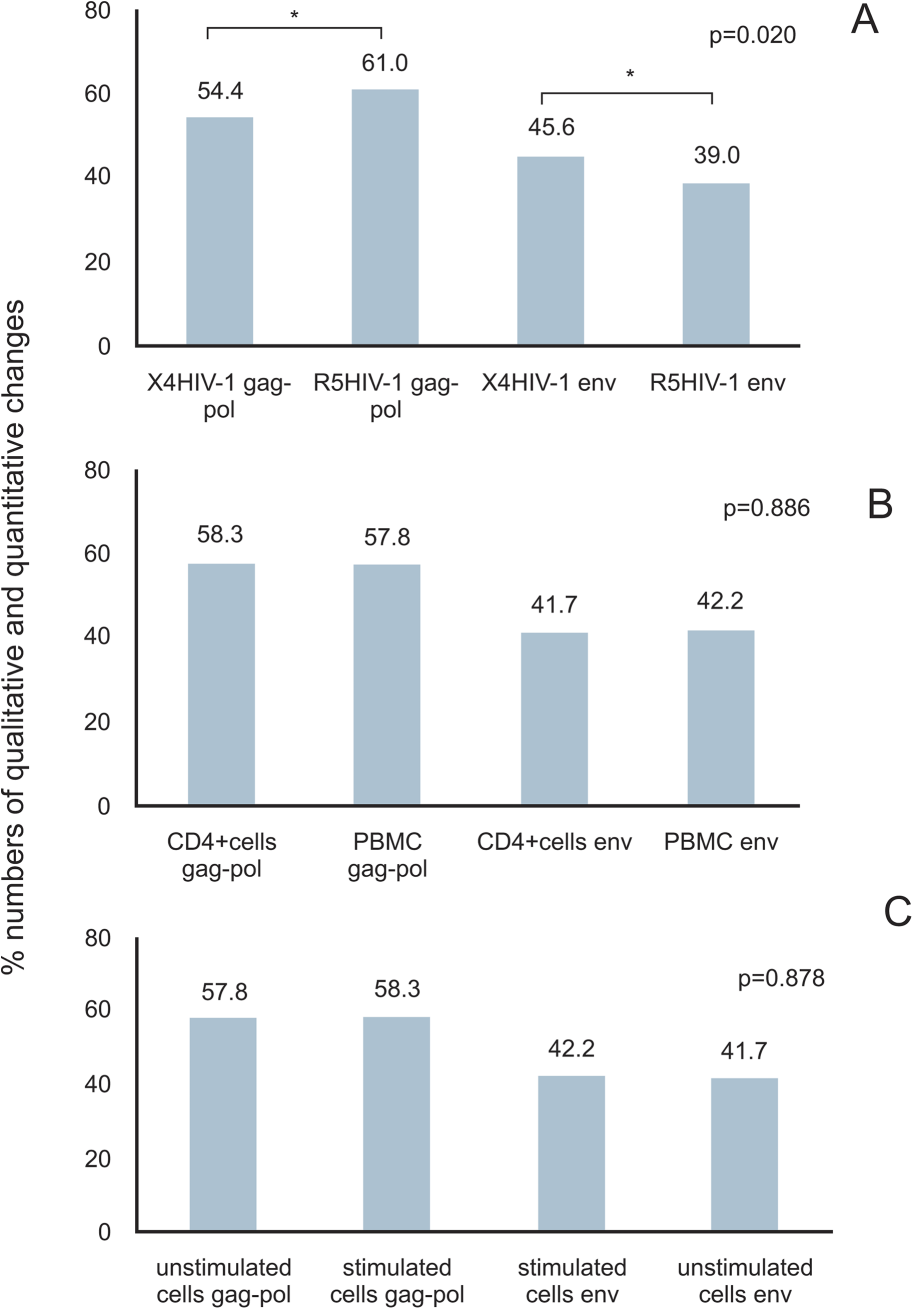
Influence of envelope tropism, cell type, and stimulation status over proviral nucleotide probabilities after one round of reverse transcription. **A)** Quantitative and qualitative changes occurring along gag-pol and env regions from X4 versus R5 pseudotyped virus infections. **B)** Quantitative and qualitative changes occurring along gag-pol and env regions from infected T CD4 + positive cells versus PBMCs. **C)** Quantitative and qualitative changes occurring along gag-pol and env regions from non-stimulated and stimulated. Asterisks indicate statistical difference at 5% of significance level between compared numbers. Probabilities are presented as percentage (%).

Data analysis allowed us to determine the probabilities of occurrence of each possible nucleotide for each position at any targeted region from the proviral DNA. Comparing nucleotide probabilities from experimental conditions to the same probabilities in the original control allowed us to identify positions with changes in these probabilities as well as the most prevalent nucleotide at each genomic position in the sequenced proviral population (see [23] for details). Since a specific pattern of differences had been identified at *env* from X4 experiments, we compared the results obtained from *env* with different genomic regions without a clearly defined pattern of probability differences as, for example, the *gag-pol* regions. This was done in order to evaluate the role of target cell type and cell activation status in generating that signature. Comparison of the variational distances and complementary probabilities revealed no statistical differences (5% significance level) when the same pseudotyped virus (either R5 or X4) infected either PBMCs or purified T CD4+ cells (Fig 5B). Furthermore, the host cell stimulation status (cell activation indirectly evaluated by phenotypic activation markers) had no statistically significant (5% significance level) impact on variational distance differences (Fig 5C).

### Interaction with CCR5 or CXCR4 did affect the nucleotide probabilities in proviral sequenced populations

Comparing nucleotide probabilities obtained from experiments in which R5 or X4 pseudotyped viruses were used, we did observe a statistically significant difference (5% significance level) between both, when compared to the initial viral genome condition prior to infections. Because all validated reads were taken into account we refer to these differences as populational changes (Fig 5A). Since virus tropism is thought to be exclusively due to the gp120 composition, our experiments were done with pseudotyped viruses differing only at their gp160 *env* product but keeping the same genome. As a consequence, the observed difference in nucleotide probabilities could be explained by cell transduction signals after CCR5 or CXCR4 co-receptor stimulation as a result of virus binding. Notably, during all infections with X4 pseudotyped virus we noted changes in the most probable nucleotide at some positions (Fig 3B and Table 5). Also, these changes occurred mostly at the *env* coding region with the exception of a single change in *pol* only observed in non-stimulated purified CD4 positive cells infected by X4 pseudotyped HIV-1 (Table 5 and S1 Data). At every X4 HIV-1 infection the most probable nucleotide after mutation was the same, and occurred at the same genomic position. In general 32% of all substitutions were transversions and 68% were transitions. The substitution A→G was the most prevalent, corresponding to 47.1% of all observed transitions (Table 5 and S1 Data). Other transitions G→A, T→C and C→T represented 23.5%, 17.6% and 11.8%, respectively. There was only one case of change in the most probable nucleotide taking into account sequenced reads from non-stimulated purified CD4 positive cells infected with R5 pseudotyped HIV-1. This change mapped at the genomic position 6438 corresponding to the *env* coding region, being the second base of the codon number 73. This position was represented by a cytosine in the control and reference strain [GenBank accession number AF324493] changing to a thymine in this experimental condition. For most of the sequenced reads in which qualitative changes were observed, a different nucleotide was observed suggesting a shift in the population at these positions.

**Table 5:** Types of nucleotide substitutions in R5 and X4 pseudotyped virus infections. Mutated positions and the frequency of replaced nucleotides in the proviral population per experimental conditions.

Mutated position	Non-stimulated CD4 + cells/ X4 HIV-1	Non-stimulated CD4 + cells/ R5 HIV-1	Stimulated PBMC/ X4 HIV-1	Non-stimulated PBMC/ X4 HIV-1
**3242**	G→T (55.1%)			
**6438**		*C→T (98*.*5%)*		
**6780**	A→C (50.9%)		A→C (51.6%)	A→C (50.7%)
**7766**	A→T (80.6%)		A→T (73.4%)	A→T (70.5%)
**7819**	*A→G (99*.*4%)*		*A→G (99*.*1%)*	*A→G (98*.*4%)*
**7842**	T→A (93.9%)		T→A (94.7%)	T→A (96.0%)
**7864**	*A→G (99*.*5%)*		*A→G (99*.*1%)*	*A→G (99*.*2%)*
**7876**	*C→T (54*.*2%)*		*C→T (50*.*0%)*	*C→T (54*.*3%)*
**7952**	*G→A (99*.*8%)*		*G→A (99*.*8%)*	*G→A (99*.*9%)*
**7977**	*G→A (99*.*7%)*		*G→A (99*.*7%)*	*G→A (99*.*7%)*
**7993**	*A→G (99*.*8%)*		*A→G (99*.*8%)*	*A→G (99*.*8%)*
**8023**	*C→T (99*.*8%)*		*C→T (99*.*8%)*	*C→T (99*.*8%)*
**8087**	A→C (99.8%)		A→C (99.9%)	A→C (99.8%)
**8091**	*T→C (99*.*8%)*		*T→C (99*.*8%)*	*T→C (99*.*8%)*
**8382**	*T→C (98*.*5%)*		*T→C (99*.*5%)*	*T→C (99*.*8%)*
**8473**	*A→G (99*.*8%)*		*A→G (99*.*7%)*	*A→G (99*.*8%)*
**8652**	*A→G (99*.*8%)*		*A→G (99*.*8%)*	*A→G (99*.*8%)*
**8711**	T→G (99.9%)		T→G (99.8%)	T→G (99.8%)
**8718**	C→G (99.9%)		C→G (99.9%)	C→G (99.9%)
**8724**	*A→G (99*.*8%)*		*A→G (99*.*8%)*	*A→G (99*.*8%)*
**8829**	*G→A (99*.*8%)*		*G→A (99*.*7%)*	*G→A (99*.*8%)*
**8871**	*G→A (99*.*7%)*		*G→A (99*.*6%)*	*G→A (99*.*7%)*
**8884**	*T→C (99*.*8%)*		*T→C (99*.*8%)*	*T→C (99*.*8%)*
**8897**	*A→G (99*.*8%)*		*A→G (99*.*8%)*	*A→G (99*.*8%)*
**8938**	A→C (99.8%)		A→C (99.8%)	A→C (99.8%)
**8978**	*A→G (99*.*4%)*	* *	*A→G (99*.*8%)*	*A→G (99*.*8%)*

Underlined = transversions, italics = transitions. Infections of stimulated T CD4 + cells and stimulated and non- stimulated PBMCs by R5 pseudotypes did not result in changes at the most probable nucleotide.

**Fig 6:**
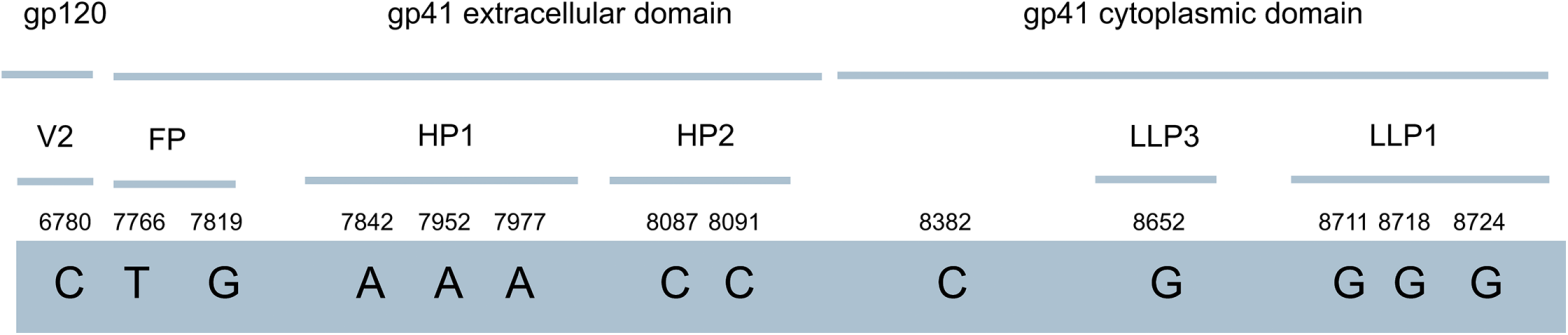
Nucleotide changes leading to non synonymous amino acids substitutions in the *env* coding region and selected known domains in the translated protein. Numbers indicate nucleotide positions. V2: variable region 2, FP: fusion peptide, HP1 and 2: heptad repeat regions 1 and 2, LLP3 and 1: lentivirus lytic peptide region 3 and 1.

Mutations occurring in the codon 73 from the *env* region along with other highlighted codons during R5 pseudotyped infections resulted in non-synonymous substitutions (Table 6 and S1 Data). In X4 pseudotyped viruses, 17 out of 25 (68.0%) mutations represented non-synonymous substitutions (Table 6 and S1 Data). A total of 13 substitutions were located in *env* and 4 in *nef* gene. Curiously, 5 out of 13 (38.0%) non-synonymous mutations in *env* occurred at places in which our control (pNL4-3kfs) differed from the reference strain NL4-3. These mutations reconstituted the original NL4-3 sequence as it appears in the GenBank (Table 6). As shown in Fig 6; 10 out of 13 (77.0%) non-synonymous substitutions in *env* could be mapped to known functional regions of gp120 and gp41 [25–28]. None of the non-synonymous amino acid substitutions in *nef* (Table 6) could be mapped to known functional domains of the protein [29, 30]. Those expected amino acid changes in *env*-*nef* gene products would be further discussed in this paper.

**Table 6:** Coding regions and mutated codons.

Coding region	Codon number	Original codon	Original amino acid	Mutated codon	Mutated amino acid	Reference strain codon	Reference strain amino acid
***pol***	386	GGG	G	GG**T**	G	GGT	G
***env***	187	GAT	D	G**C**T	A	ACC	T
	516	ATG	M	**T**TG	L	TTG	L
	533	ATA	I	AT**G**	M	ATG	M
	541	CTA	V	C**A**A	Q	CAA	Q
	548	CAA	Q	CA**G**	Q	CAG	Q
	552	AAC	N	AA**T**	N	AAT	N
	578	GTC	V	**A**TC	I	ATC	I
	586	AGG	R	A**A**G	K	AAG	K
	591	CTA	L	CT**G**	L	CTG	L
	601	ATC	I	AT**T**	I	ATT	I
	623	AAC	N	**C**AC	H	AAC	N
	624	ATG	M	A**C**G	T	ATG	M
	721	ATC	I	A**C**C	T	ATC	I
	751	TTA	L	TT**G**	L	TTA	L
	811	AAC	N	A**G**C	S	AAC	N
	831	TTA	L	**G**TA	V	TTA	L
	833	GCA	A	G**G**A	G	GCA	A
	835	TAT	Y	T**G**T	C	TAT	Y
***nef***	15	GCT	A	**A**CT	T	GCT	A
	29	GGG	G	**A**GG	R	GGG	G
	33	GTA	V	G**C**A	A	GTA	V
	37	CTA	L	CT**G**	L	CTA	L
	51	AAC	N	A**C**C	T	AAC	N
	64	GAA	E	GA**G**	E	GAA	E

Original codons are the most probable codons observed in control; mutated codons are those determined by nucleotide changes in X4 pseudotyped experiments, bold capital letters indicate base substitutions within affected codons, mutated amino acids are underlined; the 2 last columns indicate codons and amino acids at the same positions in the HIV-1 reference strain (NL4-3 GenBank accession number AF324493).

## Discussion

The two major recognized HIV-1 phenotypic variants for co-receptor use and cell tropism are relevant to the infection process and disease evolution. R5 HIV-1 is more commonly found in the beginning of infection and is thought to be the most efficiently transmitted variant. On the other hand, X4 HIV-1 is strongly associated with T CD4 positive T lymphocyte count decay, which is a hallmark towards AIDS development and a parameter for clinical evolution [2]. Besides clinical-pathological differences, biological aspects of the virus-host cell interaction have been investigated [8–10]. Our targeted population was constituted by HIV-1 provirus generated after one round of reverse transcription. The adopted m.o.i., 0.01, made unlikely the occurrence of viral multiple and super infections since there was 1 viral particle for each 100 cells in our experimental infections (see Methods section). If the infection efficiency was maximal, we could expect 10,000 single infected cells per experimental condition. Accordingly, it has been recently described the occurrence of a single copy of HIV-1 DNA per infected T CD4 positive cell in the peripheral blood [31]. Unintegrated forms are expected to be present in HIV-1 infected cells at larger amounts than provirus [32], but according to the employed DNA extraction method (see Methods section) they are unlikely to be present in the extracted material due to its small size. Our data represents a snapshot of the genetic diversity in the viral population, since the integrated form of viral genetic information is the sole genomic source for the next progeny production [33]. The magnitude of the sequencing depth coverage (Table 3) suggests that we possibly have sequenced nearly the total provirus population produced in each experiment. However, a recent report evaluating the efficacy of PCR based methods during the evaluation of microbial communities [34] showed DNA sequences representing less than 5% of the total sample tend to be poorly detected. As we did use a PCR based protocol in order to produce samples for SOLiD sequencing, it is possible we could not be able to detect some particularly minor variants in the provirus population. However, because of the extreme deep coverage of SOLiD we believe were able to describe nearly the total viral population diversity produced by the experimental infectious process.

Our data consistently showed two different patterns of nucleotide changes in provirus populations which were strongly associated with the kind of pseudotyped virus used (Figs 3–5). Our sequencing results showed a portrait of our provirus population, evidencing two different patterns of changes and showing the probabilities of occurrence of all possible nucleotides at each position in the proviral population (Tables 4 and 5). As each read is produced from a single template, the nucleotide frequency at a selected position represents the number of sequenced templates with that nucleotide. As a consequence, a difference in the nucleotide probability reflects template subpopulations in sequenced molecules harboring different nucleotides. The qualitative changes, i.e. the changes in the most probable nucleotide, occurred almost exclusively in X4 infected cells and were regularly observed at the same positions in the *env-nef* coding region taking place at the same nucleotides (Fig 3B). These results could be seen in three independent experiments with X4 pseudotyped viruses and were absent in four independent experiments with R5 pseudotyped viruses, all having the same original genomic template for reverse transcription. This suggests the stimulation of the CXCR4 pathway may influence reverse transcription or may affect a specific DNA editing activity. In support of this, previous reports in the literature [8–11] have shown that R5 and X4 HIV-1 envelopes evoke different patterns of cellular gene transcription as well as DNA editing enzyme activities. Additionally, the impact of different types of receptors in the outcome of viral infection has been recently evaluated in an engineered Moloney Leukemia Virus (MLV) model using a family of somatostatin receptors (SSTR 2, 3 and 5) [35]. MLV showed different patterns of infection, including intracellular trafficking and quantitative differences in the viral progeny related to the type of receptor employed. Despite the differences between the previously published experimental models (using for example, engineered MLV) and ours, the results demonstrated here strongly suggest that receptor types may impact the outcome of the viral diversification process. It should be emphasized that we infected cells from a single blood donor in the presented experiments, abolishing the role of different host backgrounds and reinforcing the notion of an expected outcome due to a possible cellular response. The homogeneity of our experimental procedures regarding the genetic background of cells, the types of cultured cells and culture conditions during R5 and X4 pseudotyped virus infections make the divergent results very unlikely to be related to differences arising during these procedures. Furthermore, observed differences are not likely due to the balance of nucleotides in infected cells, a parameter recognized to influence reverse transcription [36]. The direct impact of the host cell type in HIV-1 genetic variability was recently demonstrated [37]. The mutational spectra of HIV-1, in a reporter gene, was dependent on the host cell lineage and could not be attributable to the APOBEC DNA editing activity nor to the imbalance of nucleotides in infected cells [37].

In our results, the observed quantitative changes, i.e. changes in the probability of occurrence of nucleotides without changing the most probable nucleotide, occurred in both pseudotyped viruses. Cells infected with R5 viruses produced significantly more quantitative changes in the *gag-pol* coding region when compared to X4 viruses (Fig 5A). At the populational level (when all reads were taken into account), our results suggest that after one single round of reverse transcription, R5 mutations affected the balance between different subpopulations, but preserved the overall landscape of variants. On the other hand, for X4 viruses one single round of reverse transcription seems to drive a populational shift, in which specific genomic positions mutated in the majority of the proviral population (Fig 3B–Table 5). X4 viruses were less effective in generating populational diversity with changes occurring in the most probable nucleotide at some positions and resulting in a population shift towards a specific *env* region nucleotide composition. An intense HIV-1 populational genetic drift has been already observed after a single round replication in cell culture [38]. Genetic drifts are observed in small sized populations when stochastic effects help the fixation of random mutations causing the population to change haphazardly. Interesting enough the viral construction used in the above mentioned study had a CXCR4 utilizing envelope. Based on studies about RNA virus evolution, viruses may use different strategies when exploring fitness landscapes [39]. Populations of RNA viruses can act as generalists in order to survey vast extensions of fitness landscapes retaining its infectivity in different situations as for example when colonizing new hosts. On the other hand, RNA virus populations can become specialists concentrating its genetic variation in limited areas of the landscape as at the top of well defined high adaptation peaks. With this in mind we can think of R5 viruses as generalists intensifying its genetic diversity in order to explore different spaces in the landscape without concentrating its variation to defined evolutionary peaks. Since R5 using viruses are believed to be the sole transmitted variant, R5 variants are responsible for the maintenance and progression of the HIV-1 epidemic. Accordingly to our data, X4 viruses seem to act as specialists concentrating its variability to certain positions of *env* and choosing specific nucleotide substitutions. This type of evolutionary mode can be related to the appearance of X4 viruses during disease progression driving the illness to a faster pace towards immunodeficiency. In our data, changes in the most probable nucleotide occurred in the HIV-1 *env-nef* coding region. Previous reports suggest this is expected to be true in cases when natural viral evolution is observed, as opposed to certain experimental conditions in which successive bottlenecks led to unusual nucleotide substitutions in *gag* resulting in viral fitness loss [40]. The same work [40] reported the predominance of G→A transitions (43%) over other nucleotide substitution types. In our results we did not find this type of transition as the predominant one (see Results section), possibly due to the absence of APOBEC driven hypermutation (see below). They also reported [40] the absence of changes in gp120 V3 coding region, which is in accordance with our results (Fig 6). As proposed previously [40], we also associate this feature to the absence of the immune response pressure in our experimental settings. Furthermore, we analyzed a single round of infection as opposed to multiple infection rounds in the above mentioned publication [40]. In fact, the populational coverage of our sequencing data allowed us to speculate that the V3 coding region of the *env* gene did not represent a mutational hotspot after a single replication event, differently from the observed data in other viral genomic regions. That conclusion is in agreement with previous authors who suggested the hypervariability found in the V3 loop would result from positive selection instead of a particular propensity to mutate [41]. We speculate here the presence of a still unknown mechanism implicated in the generation of the viral diversity described in our results. This mechanism would act concomitantly with the initial steps of reverse transcription, after the first minus strand DNA transfer since the observed changes are strongly concentrated at the 3’ end of proviral DNA [42]. The majority of observed chances were nucleotide substitutions. Deletions and insertions occurred at very low frequencies (data not shown) and were not included in our analysis.

The different types of nucleotide substitution encountered (Table 5) argue against their appearance as a result of the APOBEC activity [12, 13]. Besides this, HeLa cells employed for pseudotyped virus production are described as not presenting APOBEC3G activity [43, 44], although the gene is efficiently transcribed [45]. The hypermutation effect of APOBEC is dependent on its transfer inside of newly assembled viral particles from the virus producing cell to newly infected cells [12, 13]. In our experimental settings there were no APOBECs being carried over inside viral particles to the next cells. In addition, the absence of detectable virus in the supernatant of infected cell cultures after the infection period (see Results section) invalidates the possibility of APOBEC carryover by PBMC or purified T CD4 positive cell-derived viruses [46]. Having this in mind, we are considering that the observed changes in the most probable nucleotide were not related to the APOBEC3G action.

The cellular activation and progress to cell cycle (Fig 1) did not have an important impact over nucleotide changes (Fig 5C). A relationship of T lymphocyte mitogen activation and increased production of cyclin B resulting in the progression of the cell cycle from G0 is referred by others [24, 47]. Cells were either kept non-stimulated or stimulated with phytohemmaglutinin and IL-2 prior to infections. The activation status was checked by flow-cytometry as shown in Fig 1. According to the MFI analysis there was an increase in the surface markers of cellular activation (CD25, CD69, CD38 and HLA-DR) when unstimulated and stimulated cells were compared. Furthermore, it has been described that HIV-1 can activate resting T CD4 positive lymphocytes at 3 days post-infection. This effect has been related to *nef* expression and evidenced by CD69 detection [48]. We could not exclude in our experiments that the unstimulated cells changed its physiological status during the infection period, following the described effect of *nef*, since we did not control the cellular activation markers expression after infection. In any case, and as mentioned before, could not see differences in viral diversity easily related to the cellular activation status.

It is accepted the existence of restriction mechanisms operating during HIV-1 infection of resting T CD4 positive lymphocytes [49], possibly: (i) at the reverse transcription step related to SAMHD1 activity which results in hydrolysis of cellular dNTP pools [50, 51]; (ii) related to cellular miRNA activity against viral and cellular gene expression [52]; or (iii) due to particularly active APOBEC3Gs [53]. In our experiments, we were able to analyze almost the complete HIV-1 coding sequence from infected cells, either without or after stimulation (see Methods section). According to our data HIV-1 infection of non-stimulated cells occurred at least until the integration step of the viral replication cycle. Infections occurred until this point regardless the cell type or viral co-receptor use. Because we used single round replication pseudotypes we can not speculate about virus production after infection of cells without stimulation. Surprisingly we could not obtain the nearly complete proviral genome sequence from stimulated purified T CD4 positive cells infected by X4 pseudotyped viruses. However it must be said that we were unable to detect viruses in the supernatant of cultures 72 hours after infection.

Target cell population homogeneity did not affect nucleotide probabilities (Fig 5B). In our experiments, we used PBMCs and T CD4 positive purified cells from the same donor in order to avoid different genetic backgrounds in host cells that could interfere with our results. In addition, by working with PBMCs we could verify the impact of different microenvironments and of bystander HIV-1 non permissive cell types (for example; negative CD4 T lymphocytes and B lymphocytes) on viral diversification. Micro-environmental conditions have been shown to influence cellular metabolism and its fate both *in vivo* and *in vitro*, through its chemical or physical components [54], in healthy [55] or pathological situations [56]. In HIV and AIDS research, this has been implicated in viral compartmentalization, which may have impact in the pathogenesis and therapeutic strategies [57]. The reasons we found similar viral diversification results with both purified T CD4 cells and PBMCs should include: (i) insufficient *in vitro* cell types compared to the blood microenvironment; (ii) little differences between PBMCs and T CD4 positive cells, regarding the cell type diversity in PBMCs; and (iiii) the virus predilection for T CD4 positive cells regardless the presence of other cell types.

The predicted amino acid changes in experiments conducted with X4 pseudotyped viruses were restricted to *env* and *nef* coding regions (Table 6). The amino acid at the position 187 of the *env* protein which is part of the V2 region of gp120 was replaced from D to A in 50% of the sequence reads [6, 58]. The V2 region along with V1 and V3 regions of gp120 are implicated in the TAD (trimmer association domain) formation which is speculated to assume different conformations according to the surrounding environment [58]. Having this in mind, one could speculate that the observed mutation in the V2 region, observed in approximately half of the sequenced provirus population (Tables 5 and 6), would be well tolerated and maintain the capability of gp120 trimmer formation. Amino acid positions 516 and 533 of *env* are part of the fusion peptide (FP) located at the ectodomain of gp41 [59]. Surprisingly the observed mutations at these positions (M516L and I533M) reconstituted the original amino acid sequence observed in the NL4-3 reference strain (Table 6). It is remarkable that over 70% of the sequenced provirus population (for position 516) and over 98% of the sequenced provirus population (for position 533) underwent this type of non-synonymous replacement. Changes at amino acid positions 541, 578 and 586 of *env* were mapped to the heptad repeat region 1 (HP1) of the gp41 ectodomain [27]. HP1 and HP2 are involved with the gp41 trimmer formation, and the observed mutations (V541Q, V578I and R586K) (Table 6) also reconstituted the original amino acid sequence observed in the reference strain (NL4-3) in more than 93%, 99% and 99%, respectively, of all sequenced reads from the provirus population (Fig 6). Albeit, changes at positions 578 and 586 resulted in the substitution of amino acids that shared the same physicochemical properties, this was not the case for position 541 in which a hydrophobic amino acid was substituted by a polar uncharged one. Changes at amino acid positions 623 and 624 of *env* were mapped at the HP2 of the gp41 ectodomain [27]. Unlike the previously described changes, these amino acid substitutions resulted in modifications in the amino acid sequence when compared to the HIV-1 NL4-3 reference strain (Table 6). At both positions over 99% of sequenced provirus had changed (N623H and M624T). Interestingly, in one these positions the replacing amino acid had similar characteristics (polar uncharged) as the observed at the same position in the reference strain (NL4-3). The change at the amino acid position 721 of *env* mapped in a gp41 unstructured region of its cytoplasmic domain [60]. The observed amino acid substitution (I721T) changed the amino acid sequence when compared to the reference HIV-1 strain in over than 98% of the sequenced provirus population, replacing a hydrophobic amino acid by a polar uncharged one (Table 6). Since this position is downstream from the transmembrane domain and out of the recognized functional domains in the cytoplasmic domain [60], it is difficult to evaluate its possible consequences. The change at the amino acid position 811 of *env* occurred at the lentivirus lytic peptide domain 3 (LLP3), and changes at amino acid positions 831, 833 and 835 at the lentivirus lytic peptide domain 1 (LLP1) of the gp41 cytoplasmic domain [26]. The mutations N811S, L831V, A833G and Y835C occurred in more than 99% of the sequenced reads in the proviral population and resulted in a modified the amino acid sequence when compared to the sequence of the reference strain (Tables 5 and 6). In the case of residues 811 and 831 the replacing amino acid shared the same physicochemical properties of the original ones. The replacing amino acid at position 833, G to A, had different physicochemical properties when compared to the original one. At position 835 the differences among replaced and replacing amino acid were significant suggesting a higher probability of conformational shift in the protein. LLP’s regions are thought to interact with the internal leaflet of the viral envelope and the cytoplasmic membrane making contact with other viral and cellular counterparts. This gp41 region is implicated in viral assembly and possibly other functions [26]. Changes in amino acids positions 15, 29, 33 and 51 mapped upstream from known functional domains of the Nef protein. Mutations A15T, G29R, V33A and N51T generated an amino acid sequence which differed from the HIV-1 NL4-3 reference strain [29]. In the case of positions 33 and 51 the substituting amino acids shared the same physicochemical properties with the original ones, while at positions 15 and 29 replacing amino acids had different characteristics. Due to their locations it is difficult to speculate on the consequences of these mutations.

## Conclusions

As a whole, our results demonstrate the feasibility of viral quasi-species evaluation by NGS methodologies.,By using SOLiD sequencing and newly developed bioinformatics analytical tools, most of the mutant spectra could be observed and analyzed [23]. We presented for the first time a strong evidence for a host cell driving mechanism acting on the HIV-1 genetic variability under the control of co-receptor stimulation. However the host mechanisms involved here are still ill defined. Further investigations are needed to clarify this question, which is relevant to viral diversification process and the consequent disease progression.

## Methods

### Plasmids, pseudotyped viruses and cells

Viruses used in this study were produced upon transfection of HeLa cells (CCL-2 ATCC-USA) with two plasmids at once, in order to obtain viral particles presenting a structural protein not coded by its genome, i.e. pseudotyped viruses. The genome encoding plasmid, pNL4-3kfs, is derived from a HIV-1 molecular clone modified to generate an *env*-defective viral genome [59]. Viruses could be rescued in the presence of an *env* encoding plasmid, co-expressed at the same cell. Plasmids pIIIenv3-1 [61] and pAD8env led to the production of NL4-3 pseudotyped with CXCR4 or CCR5 tropic envelope proteins, respectively. Pseudotyped viruses produced here were able to perform a single round replicative cycle. Virus titration was performed by b-DNA test (Chiron Corporation) according to the manufacturer’s procedure.

HeLa cells were maintained in DMEM (Life Technologies) supplemented with 10% Fetal Bovine Serum–FBS (Life Technologies) and incubated at 37°C with 5%CO_2_. Peripheral Blood Mononuclear Cells (PBMC) were obtained from a sole healthy blood donor (HIV, HBV, HCV, *T*.*cruzi* and *T*.*pallidum* negative) in accordance to the Research Ethics Committee of UNIFESP procedures (process number CEP1023/09). Ficoll-Paque (Organon Teknika) PBMC purification was performed in accordance to manufacturer guidelines with a minor adaptation: buffy-coat was laid over Ficoll instead of whole blood. CD4 positive cells were positively selected from PBMC with EasySep Human CD4^+^ Positive Selection kit (Stemcell Technologies) in accordance to the manufacturer’s guidelines. Blood derived cells (BDC)—PBMC and CD4 positive cells—viability was determined by light microscopy in the presence of Trypan Blue (Life Technologies) prior to use. BDCs were maintained in RPMI 1640 (Life Technologies) supplemented with 10% FBS or 0.1% Bovine Serum Albumin–BSA (Gibco, Life Technologies) and incubated at 37°C with 5%CO_2_.

### Transfection, cell activation, infection and flow cytometry assay

HeLa cell transfection with the suitable plasmid pairs (3 envelope: 1 genomic) was performed by Effectene (Life Technologies) according to the manufacturer’s protocol. Pseudotyped viruses were harvested 48 to 72 hours post transfection.

Prior to infection, BDCs were incubated under two different physiological conditions quiescent and cell cycle activated cells. BDCs intended to quiescent state were incubated in the presence of BSA.

Cells were stimulated with 10 μg/mL of Phytohemagglutinin (PHA) (Invitrogen) and 20 U/mL of IL-2 (Invitrogen) for 48h followed by 24h incubation with only IL-2. After this period cells were stained with anti-CD3, anti-CD4, anti-CD25, anti-CD69 and anti-HLA-DR antibodies and the cellular activation status was analyzed by flow cytometry (Vatakis *et al*., 2009). PBMCs or purified CD4+ cells without stimulus (non-activated cells) were used as control. The cell activation was analyzed on gated CD3+CD4+ cells. Data was analyzed using the FlowJo Program (Tree Star Inc.). Twenty thousand events were acquired from each gate on a FACSCanto II Flow cytometer (BD Biosciences).

10^6^ cells from every BDC group were infected with R5 or X4 pseudotyped virus, at a multiplicity of infection (moi) of 0.01. Mock infected cells were prepared for each BDC and processed as pseudotyped infected cell cultures. After an adsorption period of 2 hours, cells were washed once and new medium was added, supplemented with BSA or FBS and IL-2 according to the physiological status of the infected cells. Infected cell cultures were incubated in standard conditions for 72 hours, after which cells were pelleted down and used for DNA extraction. Supernatants were submitted to virus titration by b-DNA test.

### DNA extraction, quantification and proviral amplification

Cellular DNA was extracted with QIAamp Blood DNA Mini kit (Life Technologies) according to the manufacturer’s procedre. DNA was quantified by spectrophotometry with NanoDrop (Thermo Fischer Scientific). Nested PCR amplification of proviral sequence was performed in every DNA sample from pseudotyped or mock infected cells as well as pNL4-3kfs, with a modified complete proviral genome amplification according to a previously described protocol [62]. Original and newly designed primers (Table 7) were used to amplify four overlapping regions of the NL4-3 provirus covering positions 268 to 9527 (Fig 3B and 3C). Amplification was performed by Platinum Taq DNA polymerase High Fidelity (Life Technologies), according to manufacturer’s instructions, with 100 pmol each primer and 100–200 ng of template DNA. The cycling conditions were: (i) region “A”, both rounds, 94°C/7’; 35 cycles of 94°C/45”, 56°C/45”, 72°C/4’; 72°C/7’; (ii) region “B”, round 1 (ext primers), 94°C/7’; 35 cycles of 94°C/45”, 52°C/45”, 72°C/2,5’; 72°C/7’ and round 2 (int primers), 94°C/7’; 35 cycles of 94°C/45”, 53°C/45”, 72°C/2’; 72°C/7’; (iii) region “C” and “D”, both rounds, 94°C/7’; 35 cycles of 94°C/45”, 56°C/45”, 72°C/3’; 72°C/7’. All reactions were performed in Verit thermocycler (Life Technologies).

**Table 7:** Primers used for HIV-1 provirus nested-PCR amplification.

Amplified region	Primer name	Sense	Target position*	Primer sequence 5’-3’
**A**	1a ext	F	141 to 168	AAGTTAGTACCAGTTGAACCAGAGCAAG
	B1 ext	R	3,871 to 3,894	CCCTATTGGCTGCCCCATCTACAT
	1 int	F	268 to 290	ACAGCCTCCTAGCATTTCGTCACA
** **	B01Rint	R	3,772 to 3,791	CCACTCAGGAATCCAGGTGG
**B**	IN-B2OS ext	F	2,800 to 2,820	CTCAAGACTTCTGGGAAGTTC
	SC-BOA ext	R	5,242 to 5,267	TCTCCTGTATGCAGACCCCAATATGT
	IN-B01S int	F	3,236 to 3,258	GATGTTGTATGAACTCCATCCTG
** **	C-BNA int	R	5,193 to 5,220	CCCTAGTGGGATGTGTACTTCTGAACTTA
**C**	SC-COS ext	F	4,809 to 4,841	TACAGTGCAGGGGAAAGAATAATAGACATAATA
	SC-COA ext	R	7,821 to 7,842	TGTCTGGCCTGTACCGTCAGCG
	SC-CNS int	F	4,890 to 4,917	CAAAATTTTCGGGTTTATTACAGGGACA
** **	SC-CAN int	R	7,776 to 7,798	GCTGCCTGCTGCTCCCAAGAACC
**D**	SC-DOS ext	F	7,686 to 7,710	TTGAACCATTAGGAGTAGCACCCAC
	SC-DOA ext	R	9,514 to 9,538	AGAGAGACCCAGTACAGGCAAAAGC
	SC-DNS int	F	7,709 to 7,732	ACCAAGGCAAAGAGAAGAGTGGTG
** **	SC-DNA int	R	9,500 to 9,527	GTACAGGCAAAAAGCAGCTGCTTATATG

*HIV-1.pNL 4–3 sequence, GenBank accession number AF324493. F = forward, R = Reverse.

### PCR product purification and next-generation sequencing

PCR products of the four proviral regions from each experimental condition and control plasmid were purified with the Montage Filter PCR purification kit (Millipore), according to the manufacturer’s instructions. Polled amplified regions from the same experimental condition (30,000 ng per region) were used for fragment library production and sequencing by SOLiD v3.0 (Life Technologies), according to supplier’s guidelines.

### Next-Generation sequencing data analysis

The probabilities of the possible nucleotides (A,T,C,G) at each position of the genome are estimated from the aligned data. In this respect, the approach proposed in may be classified as a *local diversity estimation* [23]. The first stage consists in using the *experimental control data* as the input for estimation of an *a priori* distribution. The idea is that at each position in the control the probability distribution is given by a *multinomial distribution*, parameterized by probabilities (*p*_A_, *p*_T_, *p*_C_, *p*_G_) satisfying *p*_A_+*p*_T_+*p*_C_+*p*_G_ = 1. These probabilities represent the populational frequencies of each nucleotide per site. Thus, one has a family of multinomial distributions indexed by the sites of the genome. A convenient way to estimate these distributions is through the *Dirichlet distributions*.

The family of Dirichlet distributions [63, 64] is defined in terms of continuous probability densities on the set of *n*-dimensional multinomial parameters and is a multivariate generalization of a *beta distribution*–in other words, it is a family of probability densities over a set of multinomial distributions. They are characterized by a *n*-tuple of positive numbers called *hyper-parameters*. However, unlike the multinomial parameters that must sum to one, the hyper-parameters are unconstrained. In our case, the Dirichlet distributions are parameterized by a quadruple (*α*_A_, *α*_T_, *α*_C_, *α*_G_) of positive numbers. They provide maximum likelihood point estimates for the nucleotide probabilities as the mean value of the corresponding Dirichlet distributions through the formula *p*_N_ = *α*_N_/s, where N = A,C,T,G, and s = *α*_A_+*α*_T_+*α*_C_+*α*_G_ is called the *precision* of the corresponding Dirichlet distribution.

The hyper-parameters must be estimated from the data in two steps. In the first step one estimates the hyper-parameters for the *prior* distributions using the control by maximum likelihood method. In the second step, the hyper-parameters of the *prior* distributions should be used together with the sequenced data of each experimental condition in order to compute the hyper-parameters for the corresponding *posterior* conditional distributions by Bayes formula. As a result, we obtain one conditional distribution per site for every experimental condition. That enables us to obtain point estimates of maximum likelihood for each experimental condition, by calculating the mean value of the corresponding Dirichlet distribution.

Besides the nucleotide probabilities, there are two simple quantities based on them that are very useful in the analysis of the data: (i) *complementary probability per site* and (ii) *variational distance per site*. They may be used in order to establish the thresholds or to obtain some qualitative information about the behavior of the population at a site.

The *complementary probability per site* is defined as *p*_comp_ = 1–max{*p*_A_, *p*_T_, *p*_C_, *p*_G_} and it depends only on the probability distribution of each site. It provides a measure of how much the distribution is concentrated in one state. If the complementary probability at a site is high it means that there is high fluctuation at that site prior to the experiment. For instance, in a perfectly clonal population one would expect that *p*_comp_ = 0 at every site. However, the nucleotide probabilities obtained from the control are not exactly zero showing that there is some variation in the clonal population, most likely due to sequencing and PCR errors. The median of the control complementary probabilities *p*_comp_ over the whole genome can be used as a threshold for separating noise from the signal. In our experiment, the median of the control complementary probabilities is about 6.6 x 10^−4^, thus we can consider as significant any site in the experimental conditions with complementary probability above 10^−4^.

The *variational distance per site* is defined by *vd* = |*p*_A_–*p'*_A_|+|*p*_T_–*p'*_T_|+|*p*_C_–*p'*_C_|+ |*p*_G_–*p'*_*G*_|, where (*p*_A_, *p*_T_, *p*_C_, *p*_G_) is the nucleotide probability distribution per site in the control data and (*p'*_A_, *p'*_T_, *p'*_C_, *p'*_G_) is the nucleotide probability distribution of the corresponding site in the experimental condition. It is a measure of the relative variation per site from the control to the experimental data. If it is very low at a site it means that the site has not undergone significant change in relation to the control.

For more details on this method of analysis and its implementation see [23]. The complete output of the analysis applied to our seven experimental conditions and its control may be found at the S1 Data.

## Supporting Information

S1 Data Contains the data of NGS analysis of all the seven experimental conditions plus its control (ZIP). (ZIP)
